# Hydroxyl on Stepped Copper and its Interaction with Water

**DOI:** 10.1021/acs.jpcc.4c04091

**Published:** 2024-07-30

**Authors:** Kallum Mistry, Henry Snowden, George R. Darling, Andrew Hodgson

**Affiliations:** Surface Science Research Centre and Department of Chemistry, University of Liverpool, Liverpool L69 3BX, U.K.

## Abstract

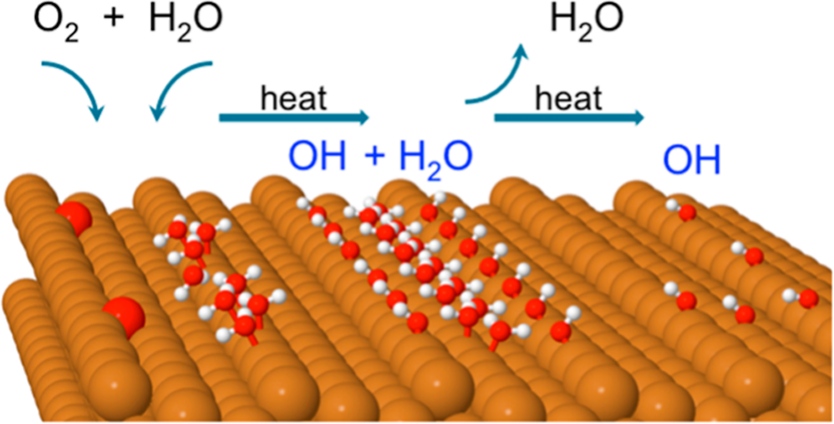

We describe the hydroxyl and mixed hydroxyl-water structures formed on a stepped copper surface following the reaction of adsorbed O with water at a low temperature and compare them to the structures found previously on plane copper surfaces. Thermal desorption profiles, STM, and low-energy electron diffraction show that water reacts with O at temperatures below 130 K on Cu(511). Two well-defined phases appear as the OH/H_2_O layer is heated to desorb excess water, a 1OH:1H_2_O phase and a pure OH phase. The 1OH:1H_2_O structure consists of 1D chains binding across two adjacent copper steps, with a double period along the step. Electronic structure calculations show that the structure has a zigzag chain of water along the terrace, stabilized by hydrogen bonds to OH groups adsorbed in the step bridge sites. This structure binds OH in its favored site and is similar to the structure observed on other open faces of Cu and Ni, suggesting that this structural arrangement may be common on other surfaces that have steps or rows of close packed metal atoms. The hydroxyl/water chains decompose at 210 K to leave OH adsorbed in the Cu step bridge site, with some forming H-bonded trimers that bridge between two Cu steps. Heating the surface causes hydroxyl to disproportionate near 300 K, desorbing water to leave chemisorbed O.

## Introduction

The presence of water and hydroxyl at catalyst surfaces is key to many commercially important reactions,^1^ but this importance is not necessarily matched by our understanding of how these species interact and bind to different surfaces. While both species can be detected at surfaces by a variety of techniques, distinguishing one from the other, or determining their binding energy and conformation, is complicated by the fragility of their hydrogen-bond structures and their sensitivity to the environment.^2^ For example, electron-induced processes can perturb the structure present^3^ or dissociate water,^4^ while the complex nature of the H-bond structures formed, which often contain a variety of different adsorption geometries in a large unit cell or a reduced dimensionality, hinder analysis using nonlocal surface structure techniques.^2^ Complementing conventional studies with low-temperature scanning probe measurements that directly image the water or water-adsorbate structure has resulted in a much more detailed insight into these phases,^5−8^ an approach that has been underpinned by electronic structure calculations.^9,10^ While STM is able to reveal the local structure of the complex or low-dimensional phases that cannot be understood from conventional techniques, atomic force microscopy can even reveal the directionality of H-bonds and local arrangement of H.^5,11^ As well as providing insights into the bonding of water, these techniques have unraveled the structure of several hydroxyl phases on metal surfaces,^10^ but few studies have examined the influence of steps.^12,13^

Copper-based materials are of particular interest, being active catalysts for existing commercial processes and for new, potentially disruptive process such as the electrochemical reduction of CO_2_, a promising route to a more sustainable production of chemicals and fuels.^14^ Water dissociation to form hydroxyl is rate-limiting in the low-temperature water–gas shift reaction, the catalyst being a Cu alloy, often Cu/ZnO/Al_2_O_3_ or other alloy,^15^ while Cu is a catalyst for the nonoxidative dehydrogenation of alcohols^16^ and Cu/Ni alloys find a role as nonprecious metal catalysts for the HOR in alkaline-based fuel cell systems.^17^ The electrochemical reduction of CO_2_ to produce methanol^18^ or more valuable C2 feedstocks is an attractive route toward sustainable zero emission fuel cycles, and selectivity toward different products is highly face-dependent on copper, with different catalyst morphologies giving control of the products formed.^19−24^ These reactions are usually carried out in alkaline conditions,^25^ for example, CO_2_ electrochemical reduction using Cu nanocubes displaying primarily (100) faces is able to selectively produce ethylene over C1 products.^26^

In this paper, we examine hydroxyl adsorption on a stepped copper surface and compare it to the adsorption behavior on the open (110) surface.^27−33^ The stepped surface chosen is the Cu(511) face, which contains narrow three-atom-wide (100) terraces separated by (111) steps, providing an ordered array of low coordination Cu sites for adsorption. We find that hydroxyl adsorbs preferentially at the bridge site on the close packed Cu steps, with some OH also adsorbed on the terrace in the form of an H-bonded trimer that bridges between two Cu steps. In the presence of molecular water, chains of a 1OH:1H_2_O structure form, consisting of an H-bonded, zigzag chain of water adsorbed on the (100) terrace between two Cu steps, donating the uncoordinated H atom to OH groups adsorbed along the Cu steps. We show that both the OH and OH/water structures are closely analogous to structures formed on the (110) faces of Cu and Ni, driven by the presence of similar low coordination metal sites to bind OH and by its ability to accept a proton from water.

## Experimental Methods

The Cu(511) sample (Surface Preparation Lab, polished to 0.05 μm and aligned <0.1°) was prepared in an ultrahigh vacuum environment (*P* < 1 × 10^–10^ mbar) by repeated cycles of Ar^+^ ion sputtering, followed by annealing to 1000 K to reorder the surface. The surface showed a sharp low-energy electron diffraction (LEED) pattern with STM imaging the steps as regular high-contrast lines spaced 6.6 Å apart. Temperature-programmed desorption (TPD) measurements for water reproduced the characteristic monolayer adsorption behavior reported previously.^34^ Experiments were carried out in two separate UHV systems: the first for TPD and LEED measurements and the second for STM imaging. For LEED and TPD, the sample was mounted directly to a liquid nitrogen cryostat via 0.3 mm diameter Ta wires that provide resistive heating, allowing the surface to be heated at controlled rates up 20 Ks^–^^1^. Water (99.9 at. % D_2_O) was degassed by repeated vacuum distillation and deposited directly on the surface using a collimated, effusive molecular beam. Adsorption or desorption was detected using a quadrupole mass spectrometer to monitor the relevant mass peak. The flux of the water beam (∼1 × 10^13^ cm^–2^ s^–1^) was determined accurately from the dose required to form the hexagonal structure that saturates the first layer on Cu(511).^35^ The water coverage is quoted as monolayers (MLs), with 1 ML defined as the density of the hexagonal water overlayer formed on Cu(511) in the absence of OH or O. Submonolayer doses of O_2_ were deposited via the same beam onto the 80 K surface and then heated to 200 K prior to the reaction with water to form OH/H_2_O films. The hydroxyl coverage present during LEED or TPD measurements is given in the same unit as that for water and is obtained from the TPD measurements based on the structural assignment of the 1H_2_O:1OH phase described later. Higher OH (or O) coverage (>0.2 ML) is estimated from the relative O_2_ dose, assuming a constant dissociation yield. LEED was used to measure surface ordering using a dual-MCP amplified LEED system (OCI),^36^ operated at <5 nA to minimize electron damage to fragile water structures.^37^

STM imaging was carried out at 80 K in a Dewar-type SPM system (Createc). The step direction was determined from the location of added Cu rows at the edge of the (511) terraces.^34^ Oxygen was predeposited on the surface at 300 K by increasing the O_2_ partial pressure in the chamber and then using STM to confirm the presence of O on the terraces. Water was deposited directly onto the surface at 80 K using an effusive (300 K) directional doser and then annealed at different temperatures to allow water to react with O and order prior to imaging. STM images of the resulting OH/H_2_O structures were recorded in a constant current mode at 80 K with an electrochemically etched tungsten tip. For surfaces with a low initial O coverage (<0.1 ML) studied here, the STM images indicate that the complete reaction of O with water to form OH occurs below 130 K. This is consistent with the rapid low-temperature reaction of O and water found on other Cu surfaces,^27,29^ but a detailed examination of the mechanism of O reaction with water^27,28^ is not considered here, where we focus on the OH/H_2_O structures formed.

Density functional theory (DFT) implemented in the VASP codes^38,39^ was used to explore and interpret the OH/H_2_O phases seen in STM images. The surface was modeled using either 5 × 2 or 4 × 2 supercells of the stepped surface unit cell, with a slab thickness equivalent to five (100) layers (120 Cu atoms for 5 × 2 supercells and 96 Cu atoms for 4 × 2 supercells) with the bottom half of the slabs fixed. The reciprocal space was sampled with 3 × 3 or 4 × 3 Monkhorst–Pack k-point sets, for 5 × 2 and 4 × 2 supercells, respectively, and a plane wave cutoff energy of 400 eV was used. Core electrons were treated with the projector-augmented wave method. As in previous work,^34,35,40,41^ exchange and correlation were included at the van der Waals corrected GGA level using the opt-B86b functional.^42,43^ All binding energies were calculated with respect to a gas-phase H_2_O molecule with isolated H atoms adsorbed on a separate slab. STM simulations were carried out using the Lorente–Persson implementation of the Tersoff–Hamann approximation.^44,45^

## Results

**Figure 1:**
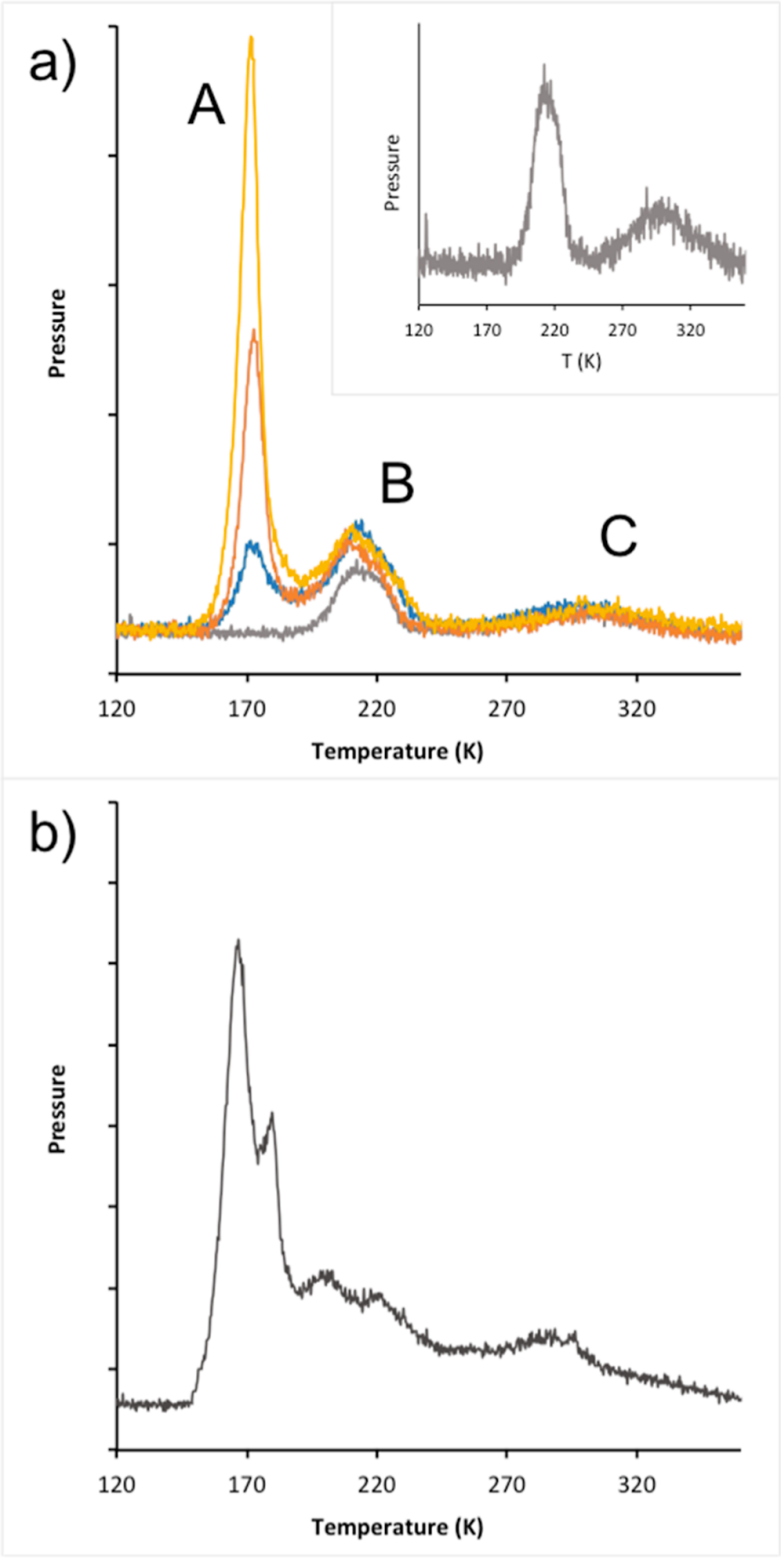
(a) Temperature-programmed desorption traces for increasing amounts of water (0.1, 0.2, 0.3, and 0.4 ML water) adsorbed on a surface pre-exposed to ∼0.065 ML of O. The inset shows an expanded view of the 0.1 ML water trace. (b) TPD trace after the reaction of 0.8 ML water with ca. 0.25 ML O showing the development of an additional overlapping structure between 175 and 270 K.

The formation of different OH/water structures by the reaction of O and water was explored using beam adsorption and TPD to establish the stability of water as a function of the amount of water and O preadsorbed. Figure 1a shows the water TPD spectra obtained when a surface covered by a small amount of O is exposed to increasing amounts of water. Three peaks appear sequentially as the water coverage increases, indicative of at least three different phases. The first feature that appears at the lowest water dose is a broad peak (C) at around 300 K, and this is followed by a narrower peak (B) near 210 K as the coverage is increased. A final peak (A) appears near 170 K when excess water is adsorbed and is similar in temperature (binding energy) to that of a pure water layer on Cu(511),^34,35^ indicating that it is associated with water that is stabilized by H-bonds to other first-layer water. Further adsorption of water caused the multilayer peak to appear near 160 K.

For low initial O coverage (less than ca. 0.1 ML), the amount of water associated with TPD peaks B and C increases linearly with the amount of O predosed. Integration shows that the amount of water desorbed in peak B saturates (in the presence of excess water) at exactly twice (2.0 ± 0.3) the amount of water desorbed in high temperature peak C, constraining the composition of these two structures. We note that the three TPD peaks have a very similar desorption temperature to those found on Cu(110),^46−49^ where the two high-temperature peaks B and C have the same 2:1 ratio^29,47,50^ as found here. In the case of Cu(110), these peaks have been assigned to the decomposition of a (1OH + 1H_2_O) structure and a pure OH phase, respectively.^29,31^ Similar TPD peaks and decomposition behavior were also observed on Ni(110)^51−53^ and are associated with the same structures as found on Cu.^54^ If we assume that the high-temperature peak C on Cu(511) also results from the disproportionation of pure OH to leave chemisorbed O and desorb water, then phase B contains one water for each OH group. On that basis, the relatively sharp peak B corresponds to the loss of water from the (OH + H_2_O) structure to leave pure OH, whereas the broad TPD peak C involves diffusion and reaction between OH groups to reform water and leave chemisorbed O. This assignment of the two phases is confirmed by the STM images and simulations described later. When the amount of O adsorbed is increased above 0.1 ML, the water TPD behavior becomes more complex, as shown in Figure 1b. Peaks B and C become less distinct and merge, with the appearance of additional overlapping TPD peaks between 175 and 210 K suggesting that a more complex mix of structures with different binding energies forms for a higher initial O coverage.

Based on the TPD results, we recorded low current LEED data to look for ordered structures that form as a function of the initial O coverage. For an initial O coverage below ∼0.1 ML, conditions where there are three well-defined peaks in TPD (Figure 1a), limited order is found in LEED, as shown in Figure S1. Half order (1/2, 1/2) beams are present when excess water is on the surface, corresponding to peak A, but as the film is heated, the beams become diffuse and elongate along the [25̅5̅] direction, indicating that a two-unit repeat persists along the step direction but the ordering perpendicular to the steps disappears as the amount of water decreases. Extended annealing to remove excess water and leave phase B causes the fractional order LEED beams to disappear, indicating that this low coverage phase has no long-range 2D order. When water is adsorbed on a surface with an O precoverage above ca. 0.1 ML, corresponding to the complex TPD trace seen in Figure 1b, broad diffraction beams appear at the 1/2 order positions for a wide range of O/water ratios and anneal temperatures between 150 and 180 K. The shape and intensity of the diffraction features indicate a two-unit repeat in both the [25̅5̅] and [01̅1] directions with a limited degree of ordering, which disappears as the surface is heated to 270 K to leave pure OH.

**Figure 2:**
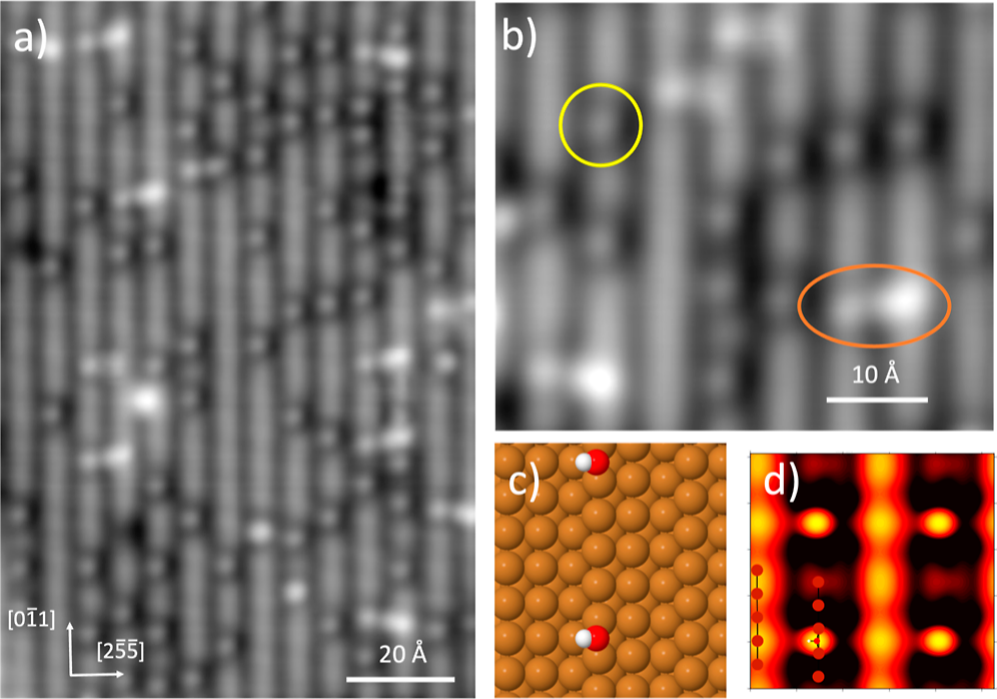
(a) STM image obtained by heating water on an oxygen precovered surface to 270 K and (b) detail showing the two types of features, an isolated feature on the step (yellow circle) and a streaked feature linking two steps (orange ellipse) (recorded at 77 K, −130 mV and 90 pA). The OH coverage is ca. 0.03 ML. (c) Calculated structure of OH showing the optimum binding site for OH from DFT and (d) its STM simulation (−150 mV).

In order to understand how hydroxyl binds on the stepped Cu surface and its interaction with molecular water, we imaged the structures found in TPD using STM, comparing these images to DFT simulations of different possible structures. We focus on the nature of the two well-defined low coverage phases, B and C, identified in Figure 1a. The copper sample was exposed to O coverage in the range 0.02 to 0.1 ML, dosed with an excess of water and then heated to 200 K to form phase C. Whereas bare metal steps image as featureless high contrast lines in STM, the Cu steps are now decorated by discrete bright features associated with OH, as shown in Figure 2. The OH groups are spaced at least two Cu atoms apart along the steps, but otherwise do not show any clear ordering along the steps. The contrast maximum sits slightly to the down-step side of the step and the contrast of the Cu step near to the OH group is reduced. In 10 to 20% of cases, we find that the contrast is enhanced and the bright feature shows a streak toward a brighter feature on the right-hand step (the adjacent up-step in Figure 2, as indicated in orange in Figure 2b). The two bright step features are slightly displaced from each other along the step, giving them the same binding site as the isolated features on both steps. We only observe these structures when the surface has been heated sufficiently to desorb excess water to leave pure OH (phase C, Figure 1), so, like the isolated features, they must be associated with OH alone. Since the 6.6 Å separation of the Cu steps is too large for two OH groups to H-bond directly to each other, some other explanation must be found.

In order to explain the STM observations, DFT calculations were performed to determine the preferred adsorption site for OH and explore the stability of small clusters. The calculations find that OH prefers to bind in the bridge site at the Cu step with the H atom pointing out across the lower terrace at an angle of ∼30° to the surface. The rotation of OH perpendicular to the step is relatively soft, and a second minimum is found with the H pointing toward the upper terrace that is just 24 meV/OH less stable. STM simulations (Figure 2d) confirm that OH suppresses the contrast of the Cu step near to OH, which images as a bright feature within a low contrast region of the step, reproducing the contrast observed in the images shown in Figure 2. The STM images indicate that OH are distributed randomly along the step but do not approach closer than the next nearest neighbor site (2a_Cu_ apart, where a_Cu_ is the atomic spacing of Cu), so binding energy calculations were performed to look at the interaction of OH groups adsorbed on a step. We find that decreasing the spacing of two OH groups has no effect until they reach the next nearest neighbor site, which reduces the binding energy by just 7 meV per OH group, comparable to the thermal energy available at 90 K. However, placing OH in adjacent bridge sites, so that they “share” one Cu atom, decreases the binding energy by 200 meV/OH, sufficient to entirely prevent OH approaching closer than 2a_Cu_. Adsorbing OH on neighboring steps has no significant effect on the binding energy, which changes by at most 2 meV/OH, consistent with the absence of any strong ordering of OH across neighboring steps. The DFT calculations also indicate that the best adsorption site on the Cu terrace is a bridge site, with H tilted to point toward the upper step, see Supporting Information Figure S2, but the binding energy is 235 meV/OH lower than on the step site. Again, a minimum is also found with the terrace OH rotated to point away from the step, being 285 meV/OH less stable than step OH.

**Figure 3:**
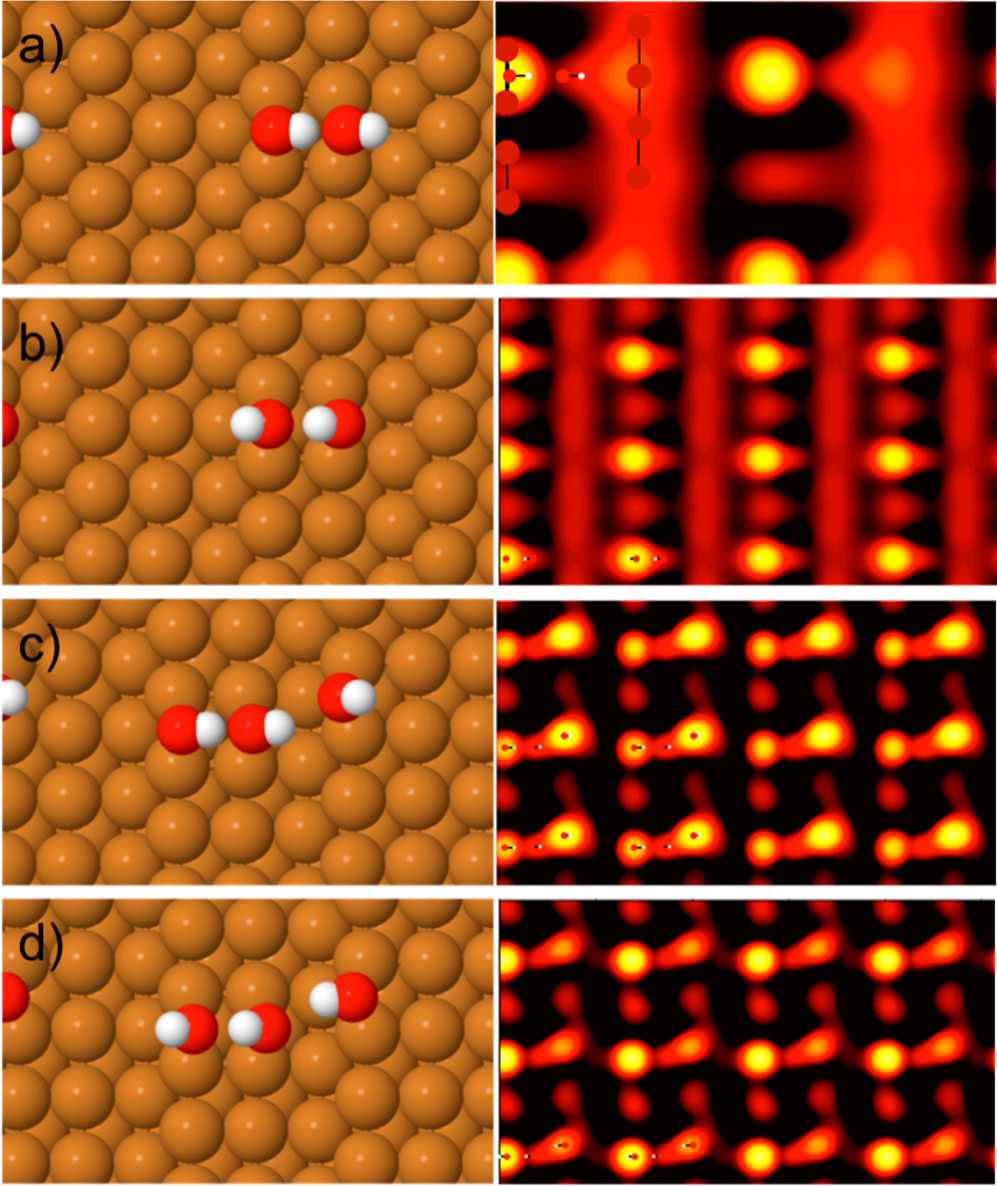
Stable adsorption geometries and STM simulations for the OH dimer (a,b) and trimer (c,d). The OH binding energies are +3, +9, −16, and −28 meV/OH greater than that of an isolated OH group, respectively. STM simulations are shown for the filled states at an energy of −0.15 eV.

To explore what might give rise to the streaked features that sometimes link two adjacent steps, we also investigated the formation of OH dimers and trimers. Figure 3 shows the structures obtained for isolated dimers and trimers, while data for extended chains are reported in the Supporting Information. The formation of a dimer requires one OH to bind on the terrace bridge site, forming an H-bonded dimer with two possible H orientations, shown in Figure 3a,b. Despite the large energy cost associated with terrace adsorption (235 meV/OH), the overall dimer binding energy is similar to that of isolated OH, being just 3 and 9 meV/OH less stable, indicating that the formation of an H-bond between the two OH groups almost exactly compensates for moving one OH from the Cu step to a terrace site. According to the calculation, the preference is narrowly to rotate the step OH group to point toward OH on the terrace, as shown in Figure 3a. STM simulations for both dimer arrangements show that all of the contrast remains on the step OH group, irrespective of the proton orientation, with the terrace OH barely visible. The lack of contrast for terrace OH means that we cannot confidently distinguish an OH dimer from a monomer by STM and so cannot be sure if OH exists primarily as a monomer, a dimer, or some mixture of the two–the isolated bright features we observe could be either. Moreover, the difference in OH binding energy between the OH monomer and dimer is small compared to the precision of DFT and comparable to the size of entropic effects (which will favor the monomer), so nor can we rely on DFT to predict which species is present.

The identity of the streaked features observed in the STM of the OH phase becomes clear when we add a third OH group to form a trimer. The final OH binds on the neighboring step bridge site, displaced 1.3 Å off the dimer axis, forming a weak (2.7 Å long) H-bond to OH on the terrace, shown in Figure 3c,d. Both possible H-bond arrangements are more stable than either the monomer or dimer, with binding energies of −16 and −28 meV/OH greater than isolated OH. STM simulations for both trimers reproduce the bright spot on the down step (LHS Figure 3) and the streak toward the up-step OH that lies slightly off the perpendicular, as seen in the STM images. STM images for these clusters have the brighter of the two features on the up-step side (right-hand side, Figure 2) of the trimer. The DFT calculations predict the OH that has an uncoordinated H atom image brightest, suggesting that the observed trimer is the structure shown in Figure 3c, with the left-hand (down step) OH rotated to form an H-bond to the terrace OH. This assignment is the opposite of what would be predicted based on the DFT energetics alone, but since the energy differences are at the limit of the precision of DFT and our STM simulations do not take into account tip interaction or zero point motion of the OH, we do not believe it is possible to decide which proton arrangement is present. Nevertheless, we can conclude that a substantial proportion of the OH on the surface is present as trimers, bridging across two Cu steps, with the remainder adsorbed as monomers or, possibly, dimers.

**Figure 4:**
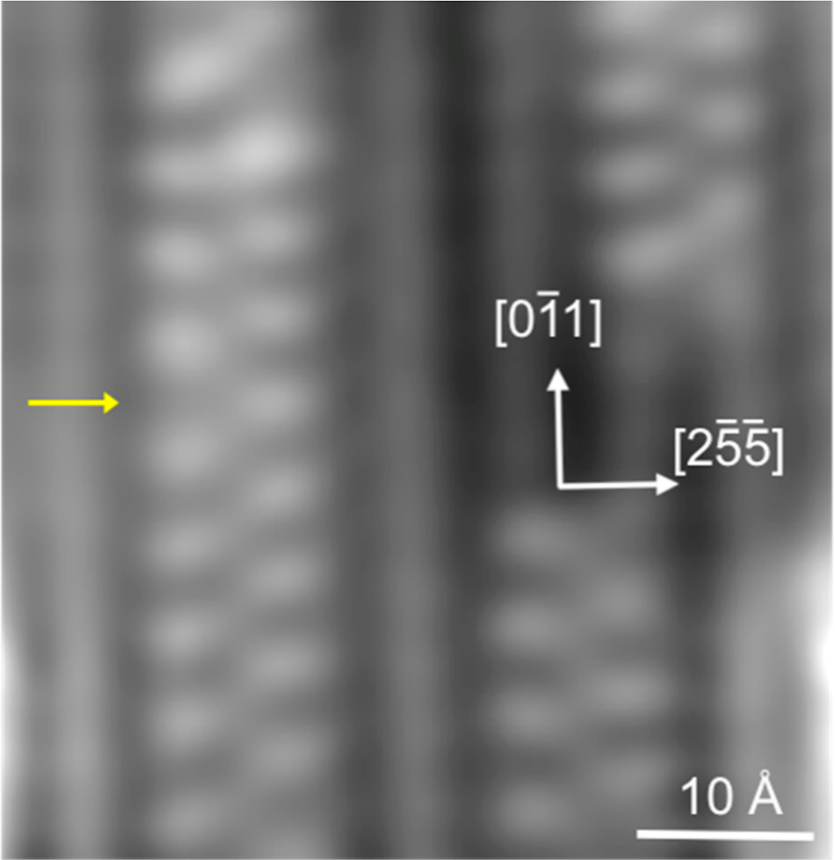
STM of the Cu(511) surface formed by preadsorbing ca. 0.05 ML O followed by water, before annealing to 170 K to remove any excess water. Zigzag chains form, aligned along the [01̅1] step direction, linking two adjacent steps. The chains have a repeat of twice the Cu atomic repeat along the steps, except occasionally on the left-hand side of the chain where a three times repeat sometimes appears, as marked by the yellow arrow. The step up direction lies from left to right, along [25̅5̅].

Figure 4 shows an STM image of the surface after depositing a small amount of oxygen, followed by excess water and annealing the surface at 170 K to form structure B (Figure 1). Extended linear features appear, aligned along the Cu step direction, appearing as zigzag structures that are insensitive to the bias voltage applied. The chains are typically between 20 and 50 Å long and consist of two parallel, staggered rows of bright features, separated by 6.6 Å, aligned along adjacent Cu steps. Each row of bright features has a 5 Å period along the steps, corresponding to twice the Cu atomic repeat, 2a_Cu_. The observation of many such chains shows that there is a difference between the regularity of the bright features on the two sides of the chain, whereas the chain on the “up-step” side of the chain (the RHS of the chains in Figure 4) is almost always spaced at exactly 2a_Cu_, the chain on the down-step side (the LHS, Figure 4) often shows errors where there is a larger (or very occasionally smaller) gap between the bright features. Most often the error is to create a single increased 3a_Cu_ spacing between the bright features on the left-hand side of the chain (for example, highlighted by the arrow in Figure 4) before the regular 2a_Cu_ repeat resumes, with the other side of the chain maintaining its regular 2a_Cu_ repeat despite the error on the left-hand side.

**Figure 5:**
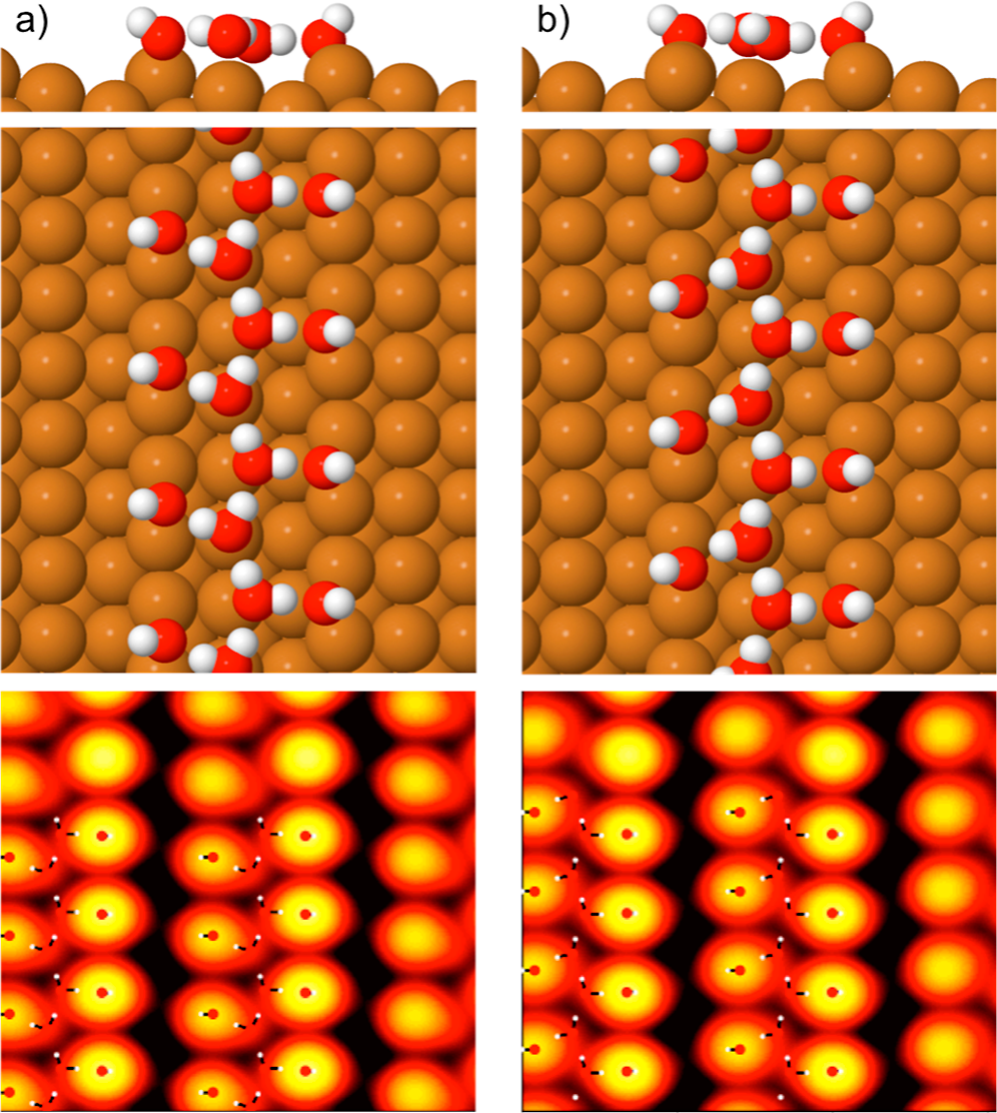
Structures for the two most stable 1H_2_O:1OH chains we found in DFT calculations, with binding energies of (a) −0.846 and (b) −0.843 eV/O atom and simulations of their filled state STM images (−0.15 eV).

From the assignment of phase C as pure OH, the TPD results define the composition of structure B as 1OH:1H_2_O. Based on this composition, we performed DFT calculations to explore the binding energy of possible structures with OH or water decorating two adjacent Cu steps and an H-bond structure linking the two steps. The two most stable 1OH:1H_2_O structures that we found are shown in Figure 5. The structure consists of an H-bonded zigzag chain of water adsorbed flat on the terrace, with each water donating one H to form an H-bond to OH at the step. This arrangement allows water to sit close to the atop Cu site, with all of the OH bound on the step bridge site. Whereas OH on the down-step side (LHS, Figure 5) points up out over the lower terrace, OH on the up-step side (RHS) has its proton direction reversed, something that costs an isolated OH just 23 meV/OH. The two structures 5a and 5b have essentially the same H-bond structure and adsorption sites and a very similar binding energy, but the OH groups on the two sides of the chain have a different phase. A slight in-plane rotation of water on the down-step side of the chain (LHS in Figure 5b) compared to structure 5a places the down-step OH in Figure 5b one unit out of phase with those in Figure 5a. Since we cannot determine the H-bond orientation of the water chain by STM, the presence of two isomers for each structure, with the direction of H-bonding in the water chains reversed, prevents us from determining which is the most common structure observed by STM. Simulation of the STM images for these two structures shows that OH images bright and the water chain has a very low contrast, consistent with the experimental images. Alternative arrangements, that have water in a two-unit zigzag, or some water and OH exchanged, are significantly less stable (see Supporting Information, Figure S4). In particular, structures that place OH on neighboring bridge sites, so that they share a Cu atom, are considerably less stable than the structures shown in Figure 5.

The identification of the two stable arrangements with OH in different bridge sites on the left-hand side of the chain helps explain the variable spacing sometimes found between OH on the down-step side of the chain (LHS). Changing one section of chain from structure 5a to 5b simply creates a single 3a_0_ wide gap between two OH groups on the down-step (LHS) of the chain without disrupting the overall H-bonding structure. This is illustrated in the chain shown in Figure 4, where the arrow shows the position of the change from structure 5a to structure 5b. In fact, calculations for short 4H_2_O + 4OH chains, where the need for a regular 2a_Cu_ repeat along the chain is relaxed, shows that a 3a_Cu_ repeat between the OH on the down step is slightly more stable than the other structures (see Supporting Information, Figure S5a).

**Figure 6:**
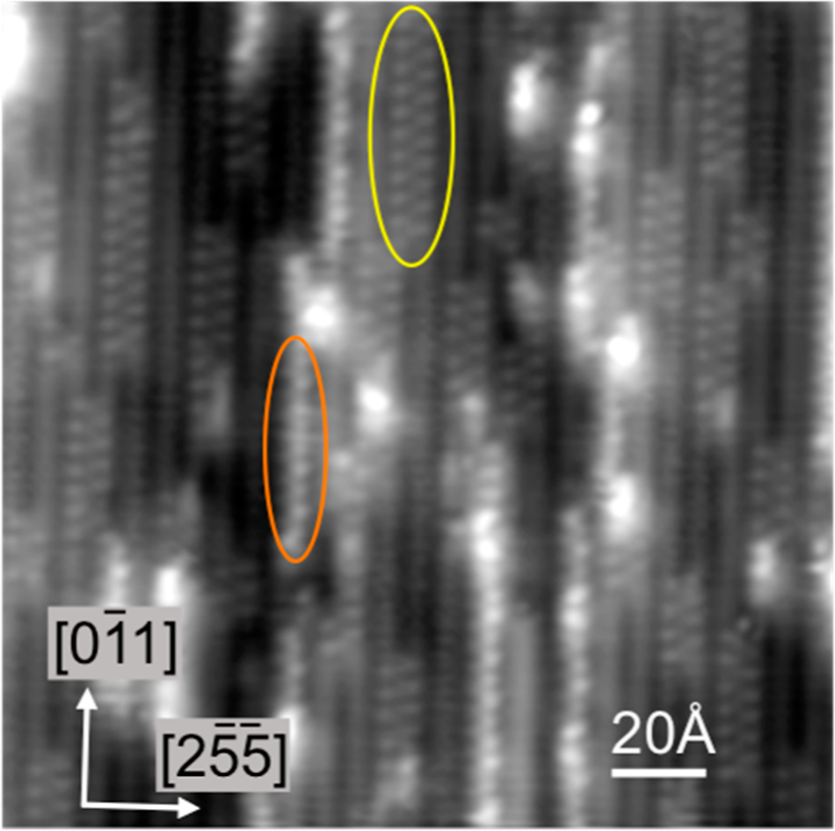
STM image of the surface following a low dose of O (ca. 0.05 ML), exposure to excess water at 80 K and annealing to 160 K. In addition to the ordered 1H_2_O:1OH chains, highlighted in yellow, bright, narrow, disordered chains appear (e.g., highlighted in orange) that run along a single Cu step.

We also used STM to examine films with a water to hydroxyl ratio greater than one, associated with peak A in Figure 1, which shows a weak (2 × 2) ordering at high coverage in LEED. However, the presence of excess water creates very disordered structures, with images showing occasional zigzag structures, similar to Figure 4, but with many bright, high contrast features and no discernible order. When a low coverage surface is annealed so that water removal to form phase B is incomplete, the surface shows additional high contrast chains along the steps, shown in orange in Figure 6. These chains are narrow compared to the two-step-wide, zigzag (OH + H_2_O) structure, but although they display a 2a_Cu_ repeat in places, the ordering is limited, and we cannot determine any single motif for these structures. Calculations show that a single chain of OH along the step with a zigzag water chain on the terrace H-bonded to the OH is only 0.052 eV/O less stable than the stoichiometric chain structure, but this arrangement (Supporting Information, Figure S4g) does not reproduce the high contrast seen in STM and the water arrangement in these narrow chains is evidently more complex and less ordered than this. In the absence of OH, water alone binds preferentially at the steps, atop Cu,^34^ but sacrifices some of the step sites in order to create a 2D network with an increased H-bond coordination. Since both OH and water prefer to bind on the step, it seems likely that these chains contain both species on the same step, with OH stabilizing the water, but the lack of order makes it difficult to learn more.

## Discussion

Hydroxyl adsorption on stepped copper displays clear similarities to the behavior on the open Cu(110) and Ni(110) surfaces, which also contain close packed rows of meal atoms with a low coordination number (7 in both cases). In each case, the preferred adsorption site for OH is the bridge site of the close packed metal row,^29,54,55^ with ESDIAD showing that the H atom is tilted over toward the surface along the [001] direction,^49,56^ making an angle of ∼40° to the surface normal on Cu(110).^31^ The close packed rows on Cu(110) are just 3.6 Å apart and OH adsorbs on two neighboring Cu rows, with one of the OH groups tilting over to form a H-bonded dimer.^29^ Dimer formation is sufficiently favorable (−0.214 eV/dimer) that no monomers are observed, but the H-bond distance is too short to allow extended chains of OH to form.^55,57^ On Cu(511), the close packed steps are 6.6 Å apart, so dimer formation would require one OH group to migrate to the terrace, and DFT calculations suggest that this process is marginally unfavorable. Although we observe what appear to be isolated features in STM, the contrast of OH adsorbed on the terrace is expected to be low, preventing us from determining if these features are isolated OH or dimers. In contrast to Cu(110), we clearly observe the formation of OH trimers on this surface. OH in the bridge site of two neighboring steps is sufficiently close to allow a third OH on the terrace to H-bond between the step OH. This arrangement provides sufficient H-bonding that it is favorable to relocate one OH group to the (100) terrace, even though OH is a weak proton donor and the H-bond distance between the terrace OH and that on the upper step is long (2.7 vs 1.9 Å for donation to the terrace OH). Displacement of OH out of its optimum molecular adsorption site in order to form an H-bond network has also been seen on other surfaces; the formation of two H-bonds from water (which is a good proton-donor) drives OH to migrate to the atop site in both the c(2 × 2) (2H_2_O + OH) network formed on Cu(110)^30^ and the hexagonal (1OH + 1H_2_O) networks formed on Pt(111)^58−60^ and Pd(111)^61^ surfaces.

The 1H_2_O:1OH chains seen when intact water is present on the surface also relate closely to the structures previously reported on both Cu(110)^29,31^ and Ni(110).^54^ On these surfaces, the 1H_2_O:1OH structure consists of a zigzag H-bonded chain of water, adsorbed flat along a close packed row of metal, with the OH groups arranged alternately down each side of the water chain accepting a H-bond from water. This arrangement allows water to bind close to its preferred atop Cu site on the Cu rows, H-bonding to OH on the neighboring close-packed metal row. The structure is stabilized by binding water and OH in their optimum metal sites (near atop and bridge respectively) while maximizing the H-bonding by completing three H-bonds per water and maximizing its donation to OH (which is a good acceptor). OH acts as a proton acceptor, but not a donor, reflecting the strong basicity of this group. STM images of all these structures image the H atoms of the decorating OH groups, the water being essentially invisible. It is not possible to construct an identical arrangement on Cu(511) as the step spacing of 6.6 Å is too large; instead, a similar structure forms with water adsorbed as a flat zigzag chain along the terrace. This arrangement sacrifices the optimum step site for single water to create an H-bond network that allows OH to adsorb at the low-coordinate step bridge sites and binds water on the terrace, close to atop Cu. Despite the LEED evidence for some preference for a (2 × 2) ordering at higher water and OH coverage, STM shows no clear evidence for any well-ordered 2D structure forming on Cu(511), which is perhaps surprising since pure water does form a stable hexagonal network.^35^ In the case of the Cu(110) surface, additional water forces OH to displace to the atop site, forming OH Bjerrum defects and maximizing the water H-bond donation to OH in a distorted 2D hexagonal network, but we cannot see any evidence this process is repeated on Cu(511), instead rather disordered OH/water structures are formed. The ability of H-bonds to neighboring hydroxyl and water groups to vary the binding site of hydroxyl, and hence the strength of the metal-hydroxyl bond, is likely to play an important role in modulating the reactivity of OH to other surface species during surface catalytic reactions.^62^

## Conclusions

Water reacts with adsorbed O at a low temperature on Cu(511), forming mixed OH/water structures that show limited order. Hydroxyl prefers to bind on the step bridge site and annealing the disordered films to desorb excess water forms a linear 1H_2_O:1OH structure based on a flat zigzag chain of water running along the terrace, H-bonded to OH adsorbed on the two neighboring steps. Heating the hydroxyl/water chains further causes water to desorb, leaving OH as either monomers or dimers with a proportion forming H-bonded trimers linking two Cu steps. The OH and 1H_2_O:1OH structures closely resemble structures found previously on the open Cu(110) and Ni(110) surfaces, suggesting that they may be common structures on other metal surfaces that have rows of low coordinate atoms.

## Supporting Information Available

LEED patterns observed, details of the DFT calculations, and binding energies of different structures (PDF)
