# Supplementary material for: Hydroxyl on Stepped Copper and its Interaction with Water
Source: J Phys Chem C Nanomater Interfaces. 2024 Jul 30;128(31):13025–33. doi: 10.1021/acs.jpcc.4c04091 (PMC11317974; doi:10.1021/acs.jpcc.4c04091)
Supplement: Supplementary file 1 — jp4c04091_si_001.pdf [file jp4c04091_si_001.pdf]

Supporting Information: Hydroxyl on Stepped Copper and its Interaction with Water

K. Mistry, H. Snowden, G.R. Darling, A. Hodgson*

Surface Science Research Centre and Department of Chemistry, University of Liverpool, Liverpool L69 3BX, UK

Supporting Figures

Fig. S1 LEED of OH/H_2O structures.

Fig. S2 DFT calculations showing minimum energy configurations for OH monomer sites.

Fig. S3 Calculated structures of OH monomer chains.

Fig. S4 Calculated structures for extended OH/H_2O chains of different composition.

Fig. S5 Calculated structures for short OH/H_2O chains.

Fig. S1

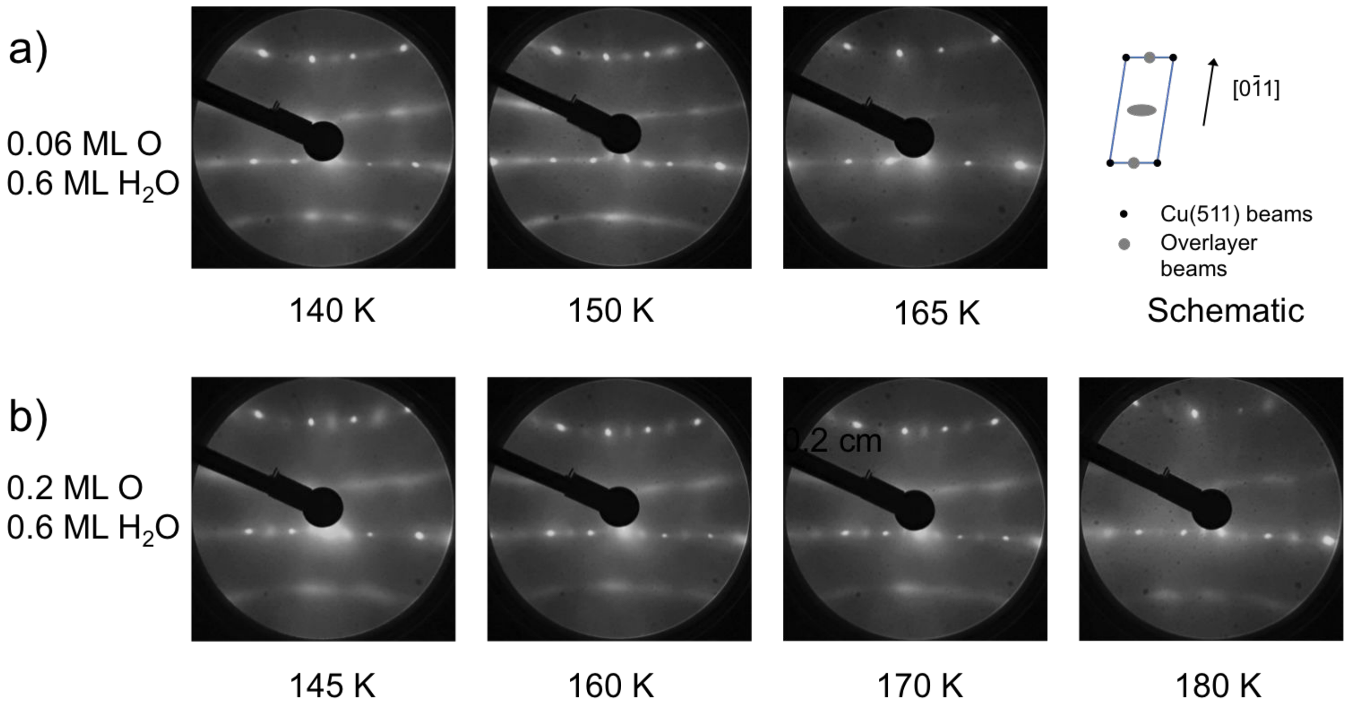

Fig. S1 LEED patterns for two different initial surface coverage of O and water showing how the LEED patterns change as the surface is heated to desorb water. In both LEED sequences the initial dose of oxygen is followed by annealing to 200 K, then cooling to 100 K to deposit water. a) shows a low coverage of O, corresponding to the situation where the $1H_2O$:1OH and pure OH phases form distinct, separate TPD traces, as shown in Fig. 1a. Initially the overlayer shows additional half order beams, indicating a double period both along the step direction and across the terraces. The order is always limited and disappears as the surface is heated to 170 K to form the $1H_2O$:1OH phase, leaving a very faint, diffuse half order diffraction feature that suggests limited two times order persists along the steps. b) LEED images for an initial O coverage of ~0.2 ML, similar to the conditions shown in Fig. 1b, again show some half order features, but now the order persists to higher temperature. At this higher O coverage some faint double period ordering persists to higher temperature along the step direction $[0\bar{1}1]$ as the pure OH and then O covered surface is formed, broadly consistent with some double period ordering of OH and O parallel to the steps at high coverage. The electron energy is 60 - 65 eV and the picture has not been corrected for the pinwheel distortion induced by the flat channel plate optics. A schematic of the substrate and overlayer beams is shown top right.

Fig. S2

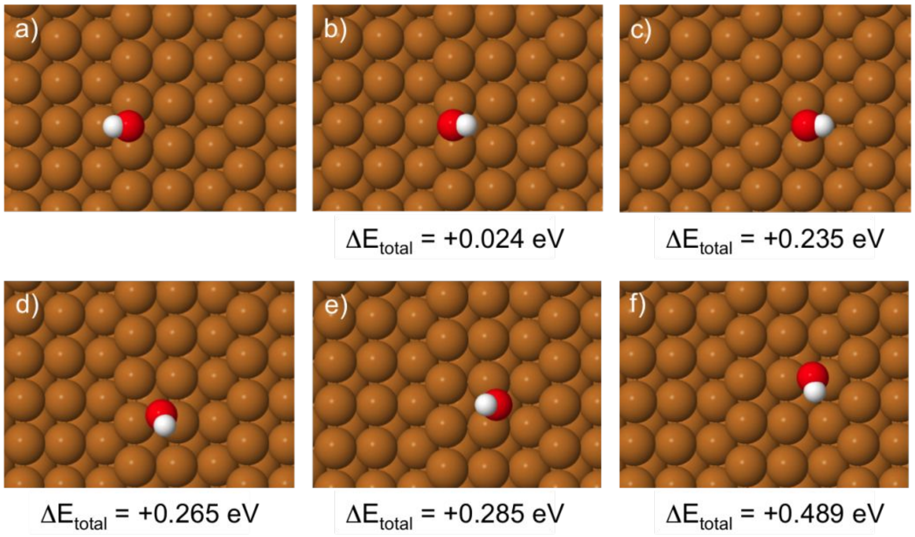

Fig. S2 Energy of different OH monomer sites on Cu(511) from DFT calculations. a) shows the optimum adsorption site with OH in the step bridge site with the H atom pointing over the down-step giving a binding energy of -0.697 eV. Subsequent frames show different minima in order of increasing energy, b) OH in the step bridge site with the H atom pointing towards the up-step, c) terrace bridge site with H pointing towards the up-step, d) bridge site above the step with H pointing along the step, e) terrace bridge site with H pointing away from the up-step and f) bridge site below the step with H pointing along the step.

Fig. S3

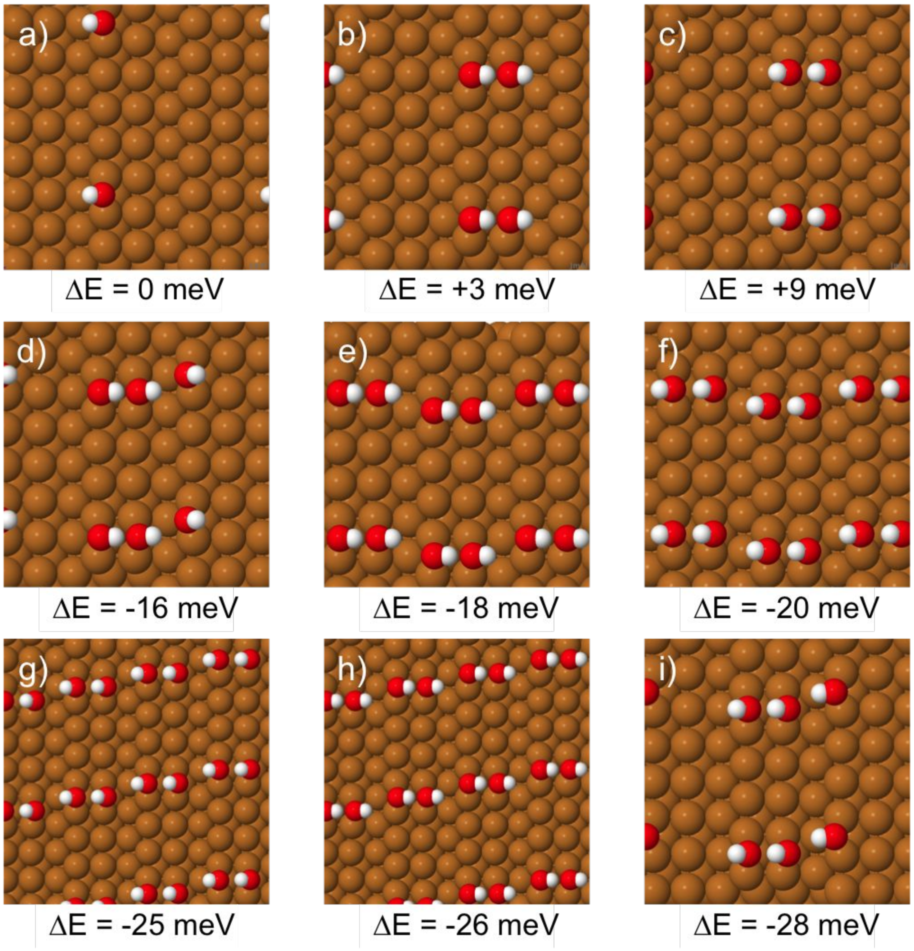

Fig. S3 Energy of different OH dimer, trimer and chain structures relative to that of an isolated OH.

Fig. S4

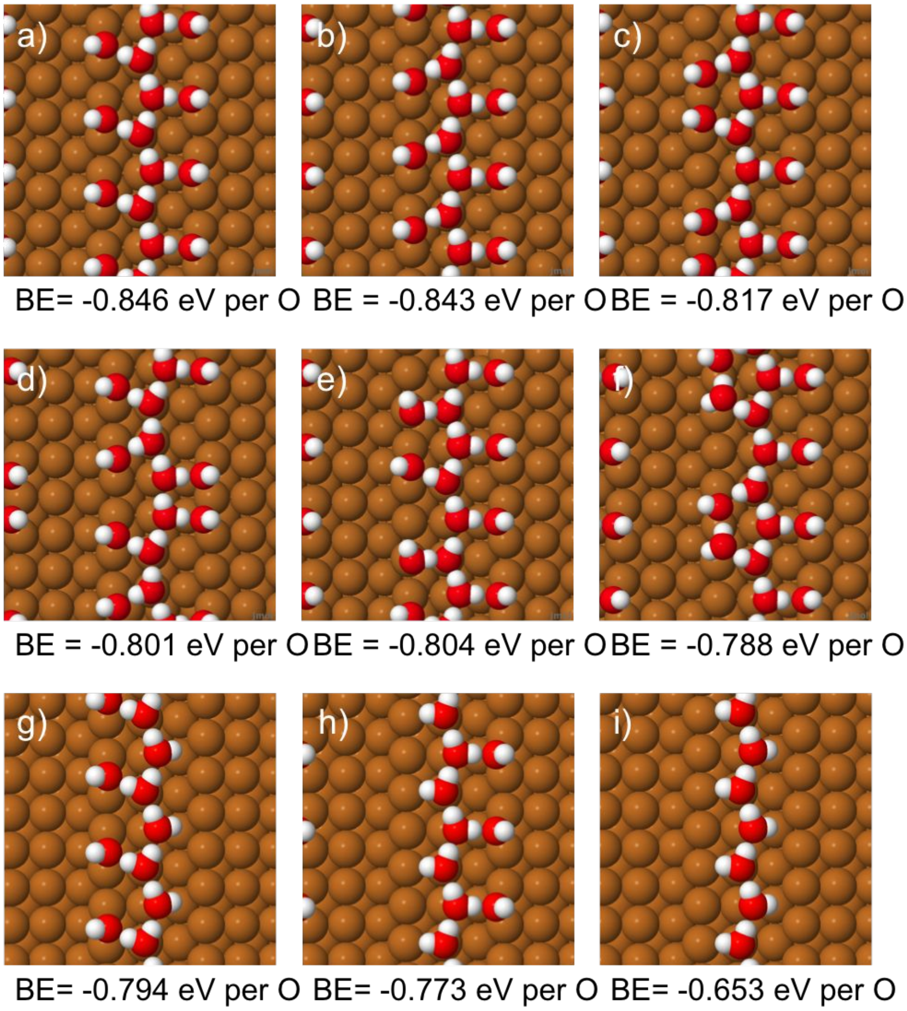

Fig. S4 Binding energy of different OH/H_2O chain structures. Frames a) and b) show the two most stable 1OH+1H_2O chains with water forming a zigzag backbone and OH bound in the step bridge site 2 Cu atoms apart. c) shows the decrease in binding energy if the OH sites on the down-step side of the chin (LHS) are given alternating 1 and 3 unit gaps. d) 1OH+1H_2O chain with the water backbone in a 4 times repeat, similar to one type of chain observed on Cu(110). e) shows the reduction in binding energy if half of the OH on the down-step side of the chin (LHS) is exchanged with water from the flat chain. f) shows the effect of replacing half of the OH groups on the down-step with water while g, h) have half the OH removed to form a 1OH+2H_2O structure. i) shows the water chain on the terrace without any OH.

Fig. S5

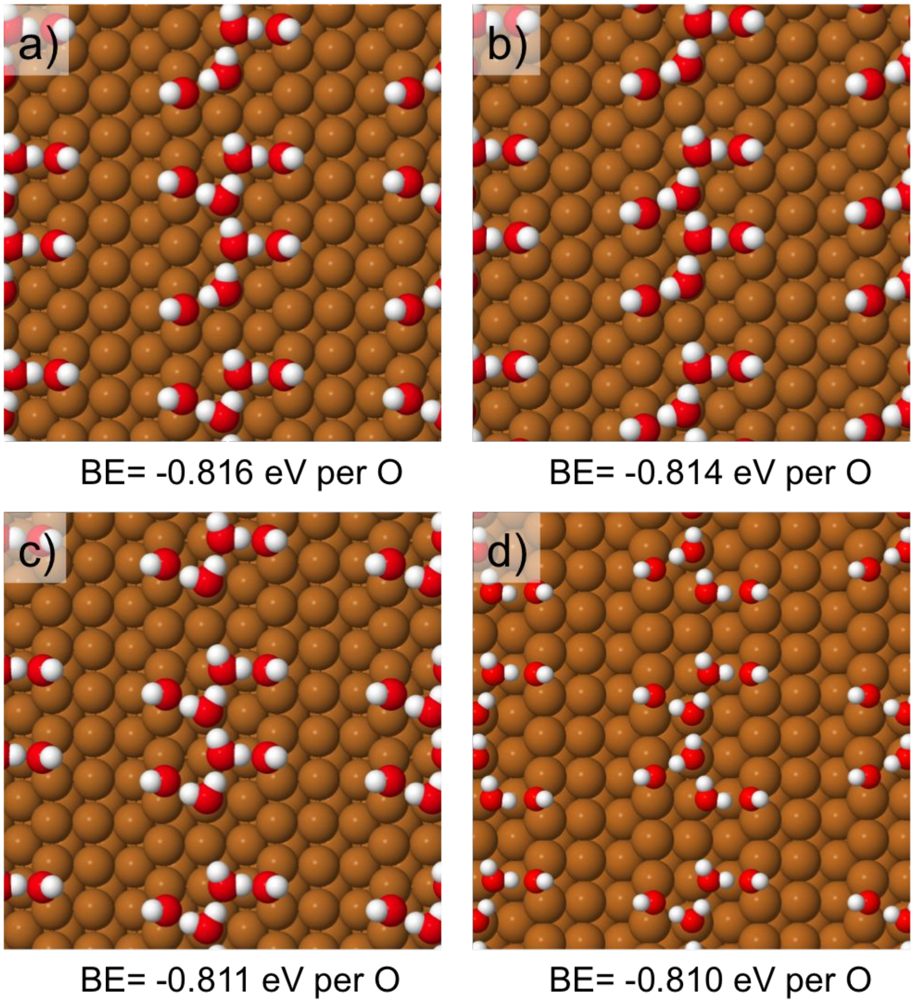

Fig. S5 Energy of short OH / H_2O clusters with different OH sites occupied. The lowest energy cluster (a) has the two OH groups on the down-step side of the chain (LHS) separated by 3 sites, indicating that the formation of this defect between structures S4a and S4b in longer chains is not expected to destabilise the chain.
